# Solvent immersion imprint lithography: A high-performance, semi-automated procedure

**DOI:** 10.1063/1.4979575

**Published:** 2017-04-03

**Authors:** S. H. Nemati, D. A. Liyu, A. J. Canul, A. E. Vasdekis

**Affiliations:** Department of Physics, University of Idaho, Moscow, Idaho 83844, USA

## Abstract

We expand upon our recent, fundamental report on solvent immersion imprint lithography (SIIL) and describe a semi-automated and high-performance procedure for prototyping polymer microfluidics and optofluidics. The SIIL procedure minimizes manual intervention through a cost-effective (∼$200) and easy-to-assemble apparatus. We analyze the procedure's performance specifically for Poly (methyl methacrylate) microsystems and report repeatable polymer imprinting, bonding, and 3D functionalization in less than 5 min, down to 8 *μ*m resolutions and 1:1 aspect ratios. In comparison to commercial approaches, the modified SIIL procedure enables substantial cost reductions, a 100-fold reduction in imprinting force requirements, as well as a more than 10-fold increase in bonding strength. We attribute these advantages to the directed polymer dissolution that strictly localizes at the polymer-solvent interface, as uniquely offered by SIIL. The described procedure opens new desktop prototyping opportunities, particularly for non-expert users performing live-cell imaging, flow-through catalysis, and on-chip gas detection.

## INTRODUCTION

Polydimethylsiloxane (PDMS) has become the workhorse material for prototyping microfluidics and optofluidics due to its low cost, simple processing, and low Young's modulus.^1–7^ However, thermoplastic polymers have recently attracted renewed interest due to their enhanced stability and low gas permeability—both particularly attractive in long-term cell culture experiments and the on-chip control of demanding chemical reactions.^8,9^ Nevertheless, imprinting thermoplastic polymers typically necessitates procedures such as hot embossing and injection molding and thus dedicated instrumentation, significant capital investments, and expertise.^10–12^ Similarly, bonding commonly requires high temperature and pressure equipment, where the optimal conditions that eliminate channel deformation^13^ and autofluorescence^14^ need to be identified *a priori*. Such dedicated, long, and expertise-intensive imprinting and bonding processes impede the wider use of thermoplastics with respect to PDMS, especially for non-expert microfabrication users.

To address these shortcomings, several benchtop prototyping methods for imprinting thermoplastic microsystems have recently been reported.^15^ In one such approach, the polymer is first dissolved in an appropriate solvent and then cast on top of a PDMS stamp, thus enabling pattern transfer from the stamp to the polymer upon solidification.^16,17^ Similarly, desktop strategies have been reported based on the use of solvent induced selective swelling in the presence of appropriate masks,^18^ directed polymer dissolution,^19^ and Shrinky-Dink based methods.^20^ However, most of the aforementioned strategies still rely on thermal fusion for bonding, while functionalizing the imprinted and bonded polymers remains a challenge. Such functionalized polymer surfaces are essential in optofluidic applications,^3,21^ whereas the microchannel regulates fluid flow, and functional moieties embedded in the channel walls act as optical sensors, or catalysts.^22–24^ In most such embodiments, the chemical moiety is first dissolved in the polymer matrix prior to the microfluidic assembly. This requirement adds an additional processing step and provides no control over the 3D functionalization architecture.^25^

We recently demonstrated *solvent immersion imprint lithography* (SIIL)^26^ to further simplify the polymer microsystem prototyping, narrow the gap between PDMS and thermoplastic processing, and enable novel 3D functionalization strategies. SIIL enables complete microsystem prototyping, including imprinting, bonding, and functionalization (or impregnation) in a single processing step with no dedicated instrumentation requirements. SIIL is based on commonly available solvents in which the polymer is immersed for a controlled duration (t_i_). During this process, the solvent penetrates the polymer forming a softened, gel-like layer at the polymer-solvent interface.^27–30^ This layer can be readily imprinted using a PDMS mask, as well as bonded to a substrate, and functionalized with chemosensing moieties. It is worth noting that SIIL's capabilities pertain not only to microfluidics and optofluidics but also to nanoscale imprinting as recently demonstrated for distributed feedback organic semiconductor lasers,^31^ and the development of modular O_2_ biosensors with the enhanced dynamic range and sensitivity.^32^

**FIG. 1.**
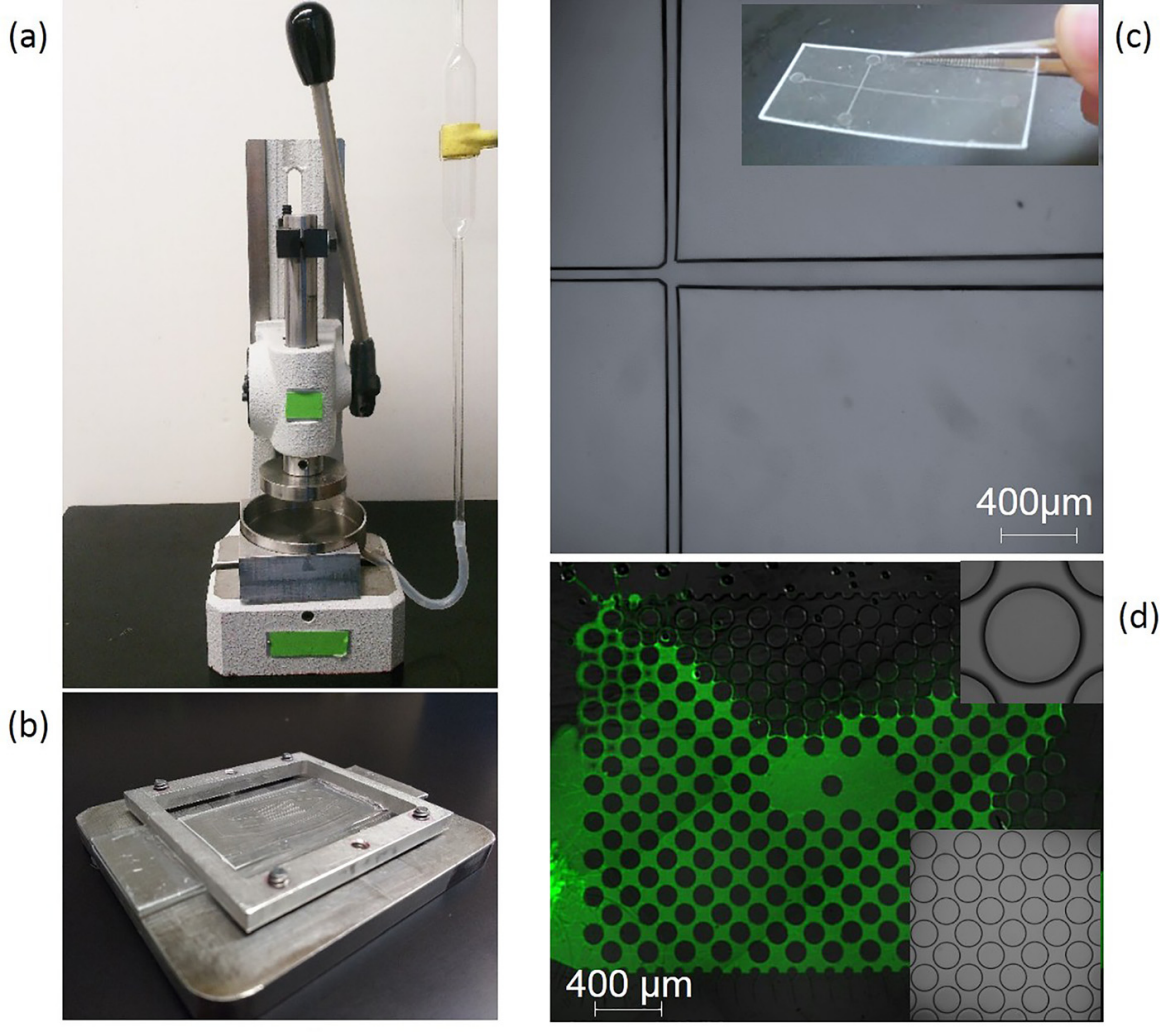
(a) The developed SIIL apparatus, illustrating the imprinting press, sample holder, and solvent control. (b) Detailed view of the aluminum sample holder. (c) An imprinted and bonded PMMA microfluidic junction for two-phase flow and droplet generation. (d) A bonded pore-scale micromodel in PMMA, in-filled with a fluorescent dye solution for visualization; inset illustrates a few select pore-throat details.

**FIG. 2.**
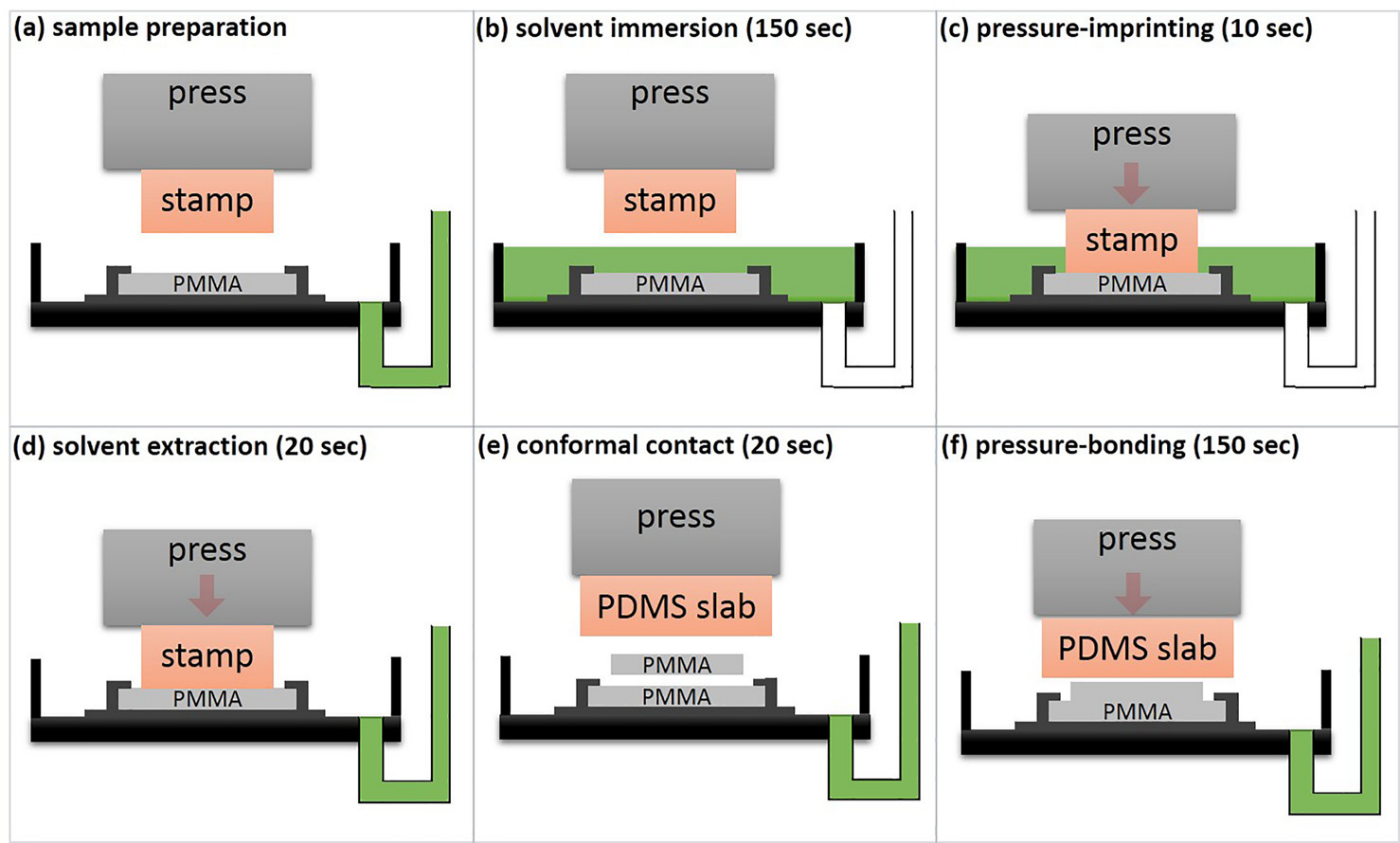
Illustration of the developed protocol, including a detailed step-by-step description.

As per our original demonstration, SIIL can be performed manually using only a beaker and a pair of tweezers;^26^ however, precision timing can greatly improve the repeatability of imprinting, bonding, and functionalization of high resolution microstructures. To this end, we report a low-cost assembly (∼$200) comprising of (1) a mechanical press (Fig. 1(a)); (2) a sample holder (Fig. 1(b)); and (3) a perforated metallic petri dish to accommodate solvent introduction and removal, through (4) a semi-automated solvent control system involving a low-cost bulb and a glass pipette (Fig. 1(a)). Using this assembly, we microfabricated a variety of microfluidic structures (Figs. 1(c) and 1(d)) and quantified SIIL's performance in spatial resolution, integration density, and bonding strength. Channels as narrow as 8 *μ*m were repeatedly imprinted and bonded with a 8 kJ/m^2^ bonding strength, more than one order of magnitude higher than previous reports on thermal and solvent mediated bonding strategies for common polymers.^8,33^

Here, our focus is on poly (methyl methacrylate) (PMMA), due to its potential in both microfluidics and optofluidics. With regard to microfluidics, PMMA is characterized by enhanced chemical inertness and minimal non-specific adsorption,^34,35^ as well as the least hydrophobicity^12^ and one of the highest electroosmotic flow mobilities^36,37^ among common thermoplastic polymers. Specific to optofluidics, PMMA exhibits a low refractive index (n = 1.48) in the visible range and thus low contrast with the refractive index of most common buffers (Δn ∼ 0.15). This in turn enables the realization of microscale optofluidic waveguides^38,39^ and the more efficient utilization of evanescent light-wave and fluid interactions.^40,41^ Furthermore, PMMA's transmission in the visible range is one of the highest among most common thermoplastics. This transmission also extends to the UV (supplementary material Fig. S1 ) and mid-infrared range (up to 4000 cm^−1^), with transmission windows of 300–1100 cm^−1^, 1350–1700 cm^−1^, 1800–2750 cm^−1^, and 3100–4000 cm^−1^ (Ref. 42)—both pertinent to resonant Raman^43^ and plasmonic sensing platforms.^44^ It is worth adding that while solvent mediated processing has been previously reported for PMMA,^19^ the procedure presented here offers significant advantages in processing duration, automation, 3D chemical functionalization, and use of cost-effective PDMS imprinting masks.^26^

## MATERIALS AND METHODS

### Polymers

PMMA films were supplied by Goodfellow (Product IDs: ME303013, ME303016, and ME303001). Different thicknesses were employed in this work, ranging from 0.25 mm to 1.2 mm, as described in the following “Protocol” and “Performance” sections.

### Solvent

Dichloromethane (DCM) was employed in all experiments (HPLC LC-MS grade—OmniSolv). The dichloromethane's Hildebrand solubility parameter is δ = 20.3 (MPa)^1/2^, moderately higher than PMMA's (δ = 19.0 (MPa)^1/2^).^45^ Such a moderate solubility difference is ideal for SIIL processing as it enables solvent diffusion and the rapid softening of the polymer matrix without inducing complete polymer dissolution.^26^ Approximately 50 ml of DCM is required for a complete SIIL imprinting and bonding process. To maintain the same processing success rate and repeatability in the procedure described below, it is recommended to exchange the solvent after 7–8 imprinting and bonding runs. As such, the processing cost is approximately $0.2 per imprinting/bonding/functionalization procedure.

### Stamps

PDMS (Sylgard 184, Dow Corning, USA) stamps were made by conventional cast-molding lithography. To this end, a monomer to catalyst solution (1:10 ratio) was thoroughly mixed, poured over SU8-on-silicon masks, and degassed for approximately 1 h prior to curing at 60 °C for 2 h. The PDMS stamps can be repeatedly used for imprinting with no visible degradation for over 100 processing experiments. However, careful cleaning with a piece of adhesive tape is recommended to remove any polymer residuals from the PDMS stamp prior to each imprinting procedure (see supplementary material (Fig. S2 ) illustrating the importance of the thorough cleaning of the PDMS stamp in imprinting and bonding polymer microchips with SIIL). In addition to the use of PDMS stamps, the PMMA films were placed on top of thin—non-structured—PDMS slabs prior to SIIL processing. This step impedes the solvent diffusion and thus the softening of the lower side of the PMMA slabs.

### Sample holder

An aluminum sample holder was employed, consisting of a rectangular plate (76 × 67 mm^2^) and a frame (54 × 54 mm^2^) (see Fig. 1(b)). The plate and frame—including any objects in-between—can be held together by four screws. A glass slide (75 × 25 mm^2^; VWR International) and a thin PDMS slab (∼1 mm thick) are subsequently placed on the plate manually (no need for O_2_ mediated bonding), followed by the PMMA film (to be imprinted). Finally, the sample assembly is placed in a metallic (petri like) dish (100 mm in diameter and depth of 20 mm). Due to SIIL's low imprinting force requirements, a glass—rather than a metallic—petri dish can be alternatively employed.

### Solvent control

To enable semi-automated solvent control, the metallic petri dish is perforated with an aperture (2 mm in diameter) in proximity to its walls. The aperture is fused with a metal tube connector extending from the bottom of the plate (∼25 mm long). A Teflon tube is in turn attached to the connector and a volumetric glass pipette (VWR) as illustrated in Fig. 1(a). The solvent from the pipette is inserted and drained from the petri-dish, controlled through a three-way suction bulb (VWR).

### Press

To enable imprinting and bonding, a low-cost press was employed (Schmidt Technology, Rack and Pinion Press Type 4). The metal dish and sample holder are placed on the base of the press, and the PDMS stamp is held with double-sided tape on the press's ram. It is recommended to maintain a PMMA film-to-stamp distance of approximately 30–40 mm prior to SIIL processing.

### Characterization

To characterize the depth and quality of the imprinted samples, we employed a profilometer (SPN Technologies Inc., Profilometer) and brightfield optical microscope (Leica DMi8 equipped with an automated stage) using 4×, 20×, and 40× magnification objectives. Brightfield optical microscopy was also employed to measure the bonding strength and area using a 4 × magnification objective.^8^ Finally, a structured illumination module (Leica Structured Illumination OptiGrid^®^) and a 20× magnification objective were employed to obtain the 3D distribution of the impregnated chromophores following SIIL processing. For this final step, a 1 *μ*m z-scanning step and a fluorescent excitation at 488 nm were chosen. To create the impregnation profile, the PMMA was immersed in a DCM solution of the Lumogen F 240 Orange dye at a concentration of 25 *μ*g/ml. All resulting images were analyzed using ImageJ (National Institutes of Health).

## PROTOCOL

A comprehensive protocol for SIIL processing imprinting, bonding, and functionalization is listed below and graphically illustrated in Fig. 2:

1. *Preparation* (Fig. 2(a)): PMMA films are cut into areas equivalent to the employed PDMS stamp—approximately 25 × 45 mm^2^. Typically, we employ 1.2 mm thick PMMA pieces for imprinting and thinner ones for bonding (typically 0.25 mm)—unless stated otherwise. The thin PMMA films are cut with a pair of scissors; thicker pieces are cut with a surgical scalpel. The PDMS stamp is thoroughly cleaned with an adhesive tape prior to each individual use. Subsequently, the PMMA film is securely mounted onto the sample holder, and the PDMS stamp placed on the press's ram using a double-sided tape. The PDMS stamp is aligned with the PMMA film, followed by the in-filling of the volumetric pipette with approximately 50 ml of DCM. All solvent mediated experiments were performed in a chemical fume hood.
2. *Imprinting* (Figs. 2(b)–2(d)): The DCM is introduced into the petri dish using the three-way pipette bulb; following complete PMMA immersion in DCM, the polymer is kept immersed for 150 s (Fig. 2(b)). Subsequently, the PDMS stamp is pressed against the immersed PMMA for 10 s (Fig. 2(c)). Longer imprinting durations were not found to affect the size/depth of the imprinted features as detailed in the “Performance” section. A detailed analysis of the imprinting force requirements is included in the “Performance” section. Still under a constant pressure, the solvent is removed using the pipette bulb, and pressure continues to be exerted for another 20–30 s (Fig. 2(d)). The latter is a critical step for the polymer to retain some small amount of solvent for bonding.
3. Following imprinting, the press ram is released to remove the PDMS stamp from the imprinted PMMA film. A non-treated PMMA film (with similar dimensions) is immediately applied on top of the imprinted layer, following manual alignment. To enable microfluidic interconnection, the non-treated piece is perforated with holes at desired locations using a 21 G syringe needle or a mechanical drill. For successful bonding, care needs to be taken to achieve a conformal contact between the non-treated and imprinted PMMA pieces and remove all air in-between. Following the contact between the two PMMA films, a non-structured PDMS slab is placed on top of the assembly and pressure—equivalent to the pressure exerted during imprinting—is applied for 150 s (Fig. 2(f)).
4. *Functionalization*: For prototyping functionalized polymer microfluidics and optofluidics, steps 1–3 are repeated where a solution of the desired chemical moiety is employed instead of the pure solvent. In the experiments described here, Lumogen F 240 Orange was employed for visualizing the impregnation architecture.
5. *Completion*: Following bonding, the press is released and the sample holder is removed outside the metallic dish. The PMMA microfluidic chip is carefully removed from the sample holder by releasing the four screws.

## PERFORMANCE

To analyze the SIIL performance via the aforementioned protocol, we characterized parameters critical to the assembly of microfluidic and optofluidic microsystems, namely, (1) imprinting depth, (2) imprinting quality, (3) bonding strength, and (4) impregnation characteristics.

### Depth

**FIG. 3.**
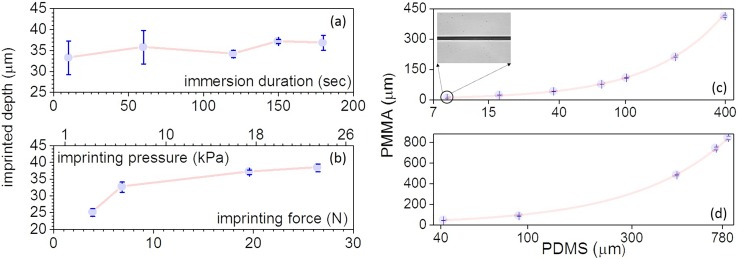
The dependence of the imprinted depth as a function of the immersion time (a) and imprinting force (b). For these, a stamp with a 100 *μ*m wide and 46 *μ*m deep microchannel was used. (c) The relationship between the microchannel dimensions in the PDMS mask (x-axis) and in PMMA (y-axis): the x-axis is represented in log-scale for visualization; and the inset shows a micrograph of an 8 *μ*m wide microchannel. (d) The same as (c), but describing the integration density, namely, the minimum channel-to-channel separation that can be repeatedly imprinted. Both experiments described in (c) and (d) were fit with a linear equation, and found to exhibit the slopes of 1.036 and 1.02, respectively. All data represent the average of three measurements, with the error bars denoting the standard deviation. For both (c) and (d), the stamp's features were 46 *μ*m deep.

In SIIL, the imprinting depth can be regulated through the height of the stamp's features and imprinting force (F_i_), provided that the interfacial gel has reached an adequate depth.^26^ As we have previously analyzed, the gel depth depends primarily on the underlying mesoscale polymer-solvent interactions and thus on the immersion duration (t_i_) and the polymer and solvent selection. For PMMA immersed in DCM, as well as identical imprinting forces of 19.6 N (or 17 kPa) and PDMS stamps, we found that immersion durations ranging from 10 s to 180 s resulted in the same imprinting depths of 35 *μ*m ± 2 *μ*m—comparable to the height of the stamp's features (46 ± 2 *μ*m) (Fig. 3(a)). This finding evidences the generation of a surface gel layer of constant depth for all the aforementioned immersion durations, as typically expected for type II Fickian diffusion during solvent immersion.^46^ Thinner imprinted features were possible (∼5 *μ*m) by selecting stamps with features of comparable heights. Similar to our previous work,^26,45^ thicker features (60 *μ*m–80 *μ*m) can be attained by selecting solvents with Hildebrandt parameters closer to PMMA, such as chloroform (δ = 18.7 (MPa)^1/2^). Unlike the immersion duration (t_i_), the magnitude of the imprinting forces (F_i_) was found to regulate the imprinting depth, as illustrated in Fig. 3(b). However, the experimentally determined force-depth curve is non-linear, which is indicative of the PMMA gel behaving as a non-Newtonian shear-thickening fluid under pressure^44^ (Fig. 3(b)). For this reason, it is recommended that potential users perform similar calibration curves prior to selecting the imprinting forces as a means to regulate the imprinting depth. Overall, the investigation revealed that SIIL requires imprinting forces less than 20 N (approximately 17.8 kPa). Such force magnitudes are substantially lower than those required for thermal imprinting and hot embossing,^47^ making SIIL ideal for use with soft and non-planar stamps.

### Quality

To determine the imprinting quality, we investigated the imprinting resolution and integration density, namely, the minimum feature size and minimum channel-to-channel separation that can be repeatedly imprinted on chips. We found that microchannels down to 8 *μ*m (∼10 *μ*m deep) could be readily imprinted and bonded, as shown in Fig. 3(c). Features smaller than 8 *μ*m could be imprinted but suffered from reduced repeatability (<70%). This size limitation is attributed to the reduced mechanical stability of the PDMS stamp, especially for aspect ratios equal to and greater than 1:2. With regard to the integration density, the smallest separation distance between bonded microchannels that we could repeatedly obtain was approximately 40 *μ*m (Fig. 3(d)). The imprinted feature size and feature-to-feature spacing exhibited a 3.6% and a 2% increase in comparison to the PDMS stamp, respectively (Figs. 3(c) and 3(d)). This value was determined by the linear fits illustrated in Figs. 3(c) and 3(d), and is attributed to minor swelling effects of the PDMS stamp during the imprinting process.

### Bonding

**FIG. 4.**
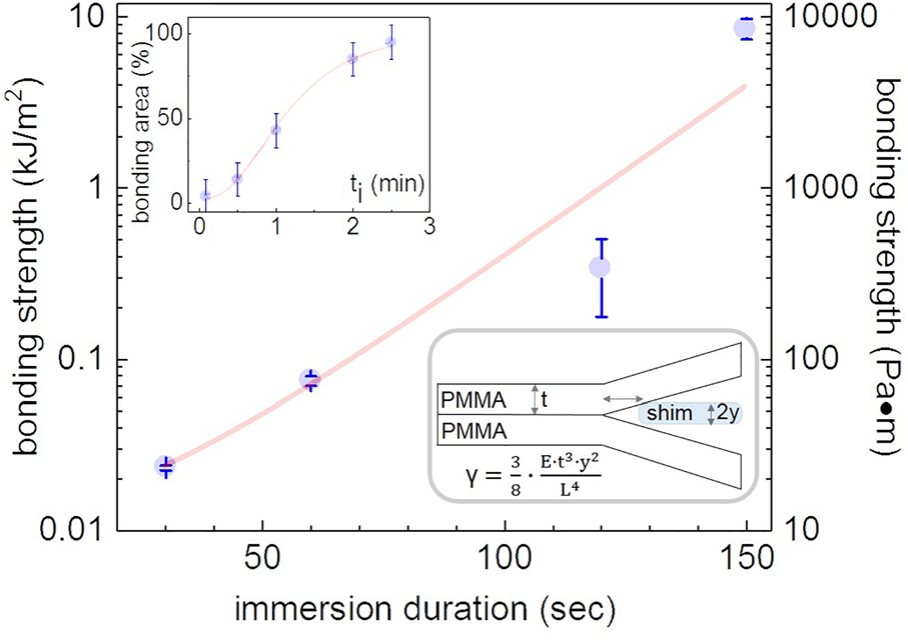
The dependence of the bonding strength on the immersion duration for PMMA in dichloromethane (DCM) performed in duplicate. Insets plot the bonded area coverage (*top*) and illustrates the experimental apparatus and model used in the experiment (*bottom*). With regard to the model parameters ,“E” is the elastic modulus of PMMA, “L” is the distance between the leading edge of delamination and the shim, while the rest of the model parameters are depicted in the schematic of the experimental apparatus.

To characterize the bonding strength, we employed the “wedge method,” as previously described.^8,33^ Briefly, we inserted a 73 *μ*m thick metallic shim between two bonded polymer films (see inset in Fig. 4), and determined the distance between the leading edge of delamination and the shim itself by optical microscopy.^8,33^ Subsequently, we derived the bonding strength (γ) from the distance measurements, using the value of 2.85 GPa for the PMMA's elastic modulus (according to the manufacturer) and the equation shown in the inset of Fig. 4.^8,33,48^ Our results indicated that longer immersion durations enabled the assembly of microchips with increased bonding strengths. Specifically, for a 150 s immersion, the bonding strength was approximately 8 ± 1 kJ/m^2^ (8 ± 1 kPa m) (Fig. 4). In addition, for a 150 s immersion duration, the bonded area was greater than 94%, as shown in the inset of Fig. 4. The reported bonding strength value is more than one order of magnitude higher compared to solvent or thermally bonding PMMA or polystyrene (PS) films, as has been previously reported by Wan *et al.* and Young *et al.*^8,33^ We attribute the increased bonding strength to the highly controlled polymer dissolution during SIIL, which is strictly localized at the polymer-solvent interface. Following imprinting, some solvent is retained at the polymer—air interface, enabling solvent exchange between the imprinted and planar PMMA films upon contact. Such strictly interfacial polymer surface softening is challenging to achieve with conventional thermal and previously reported solvent mediated bonding schemes.^15^

### Functionalization

**FIG. 5.**
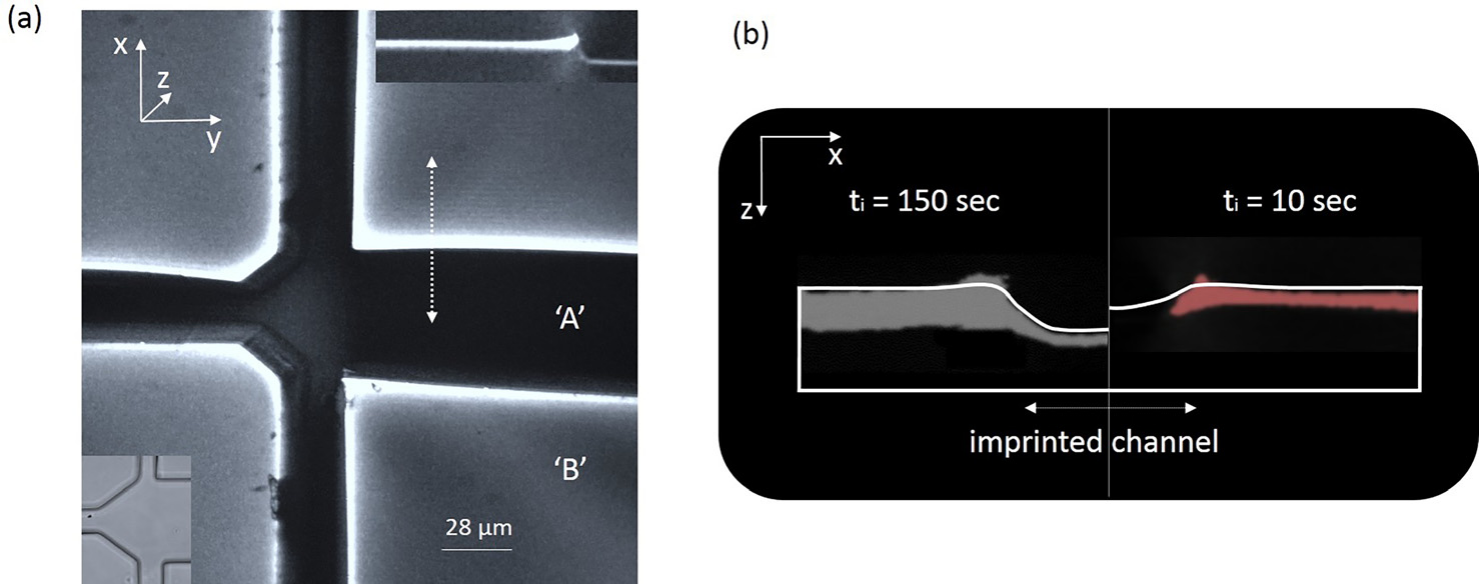
(a) A 3D fluorescence image of an imprinted and impregnated microchannel in PMMA (the same imprinting pattern as illustrated in the micrograph of Fig. 1(c), following immersion in a dichloromethane solution for 150 s. The *top* inset plots a cross-sectional view of the same channel along the dotted arrow (i.e., along the x-z plane), and the *bottom* inset plots the bright-field transmission image of the same structure. (b) A panel comparing the impregnation profile for two different immersion durations: t_i_ = 150 s (left—grey) and t_i_ = 10 s (right—red). The profiles are plotted depthwise along the channel in the x-y plane (the same orientation as in (a)). The solid white lines denote the imprinted and non-imprinted areas of the polymer matrix. The noticeable bumps at the edges of the channel walls are due to the elevated fluorescence intensity at the channel-wall edges, in turn due to elevated dye concentration levels

To assess SIIL's functionalization capabilities using the new protocol, we immersed the PMMA in a DCM dye solution rather than a pure solvent. Subsequently, imprinting and bonding were performed with 24 N forces (21 kPa) and 150 s immersion durations, as previously described. Following their assembly, the chips were imaged using a 3D confocal microscope (see “Materials and Methods” section). Similar to our previous demonstrations,^26^ this procedure resulted in a 3D impregnation architecture, by functionalizing both the walls and the bottom surface of the microchannel (illustrated as “A” and “B” in Fig. 5(a)). This form of functionalization conveniently enables the modular on-chip detection of gases,^49,50^ as well as the independent control of the sensor's sensitivity and dynamic range as we have previously shown.^26^ 3D functionalization profiles were possible to attain for immersion durations longer than 60 s, as shown in Fig. 5(b) and in the supplementary material (Fig. S3). Shorter immersion durations rendered a 2D impregnation profile (Fig. 5(b)) by impregnating only the microchannel walls (“B” in Fig. 5(a)). This evidences that the underlying solvent and dye molecules do not exhibit the same transport kinetics during solvent immersion.^32^ Therefore, we recommend potential users interested in polymers other than PMMA to first analyze the microchip functionalization architecture, in order to identify the optimal immersion durations for their applications and selected solvent-polymer-dye combinations.

## CONCLUSIONS

We reported a comprehensive and semi-automated procedure for SIIL prototyping of thermoplastic microsystems. By employing a low-cost assembly (∼$200), imprinting, bonding, and functionalization of polymer chips are possible at processing cost and duration of less than $0.2 and 5 min per chip, respectively. We characterized the SIIL's performance in imprinting quality and bonding strength using PMMA as a model polymer matrix. The analysis indicated that high resolution is attainable at substantially reduced imprinting force requirements and a 10-fold increased bonding strength compared to thermal methods. We attributed this enhanced performance to the highly controlled interfacial polymer-solvent interactions that are intrinsic in SIIL; the described procedure opens up new high-performance, cost-effective, and strictly desktop possibilities of polymer microsystem prototyping.

## SUPPLEMENTARY MATERIAL

See supplementary material for Figs. S1–S3.
